# Integrative single-cell and bulk RNA sequencing unravels the role of ACTN1 in promoting lung cancer with brain metastasis and epidermal growth factor receptor-tyrosine kinase inhibitor resistance

**DOI:** 10.3389/fcell.2026.1738641

**Published:** 2026-04-14

**Authors:** Wentian Wu, Min Yang, Jiaxuan Qin, Shangjia Gui, Ziyu Zhang, Yiruo Zhang, Yingying Du

**Affiliations:** 1 Department of Oncology, The First Affiliated Hospital of Anhui Medical University, Hefei, China; 2 The First Clinical School of Anhui University of Traditional Chinese Medicine, Hefei, China; 3 Department of Urology, The First Affiliated Hospital of Anhui Medical University, Hefei, China

**Keywords:** brain metastasis, EGFR-TKI resistance, lung cancer, machine learning, single-cell RNA sequencing

## Abstract

**Background:**

Brain metastasis (BM) remains a severe and fatal complication in patients with lung cancer (LC), presenting a major therapeutic challenge. Although epidermal growth factor receptor-tyrosine kinase inhibitors (EGFR-TKIs) have emerged as a cornerstone of targeted therapy, their clinical efficacy is often limited by the inevitable development of drug resistance.

**Methods:**

We initially constructed a general atlas of the tumor microenvironment (TME) in LCBM lesions by integrating single-cell RNA sequencing (scRNA-seq) data. The sensitivity of each cell cluster to EGFR-TKIs was assessed by the “Beyondcell” method. By performing high-dimensional Weighted Gene Co-expression Network Analysis (hdWGCNA), we identified hub genes within an EGFR-TKI resistance-associated cell cluster. Finally, the functional role of the most promising candidate, ACTN1, was further investigated in a constructed osimertinib-resistant LC cell line.

**Results:**

We identified a malignant and therapy-resistant ACTN1^+^ epithelial cell subcluster. Both signaling and functional enrichment analyses demonstrated marked activation of PI3K-Akt and IL-17 signaling pathways in ACTN1-high patient subgroups. Finally, we applied machine learning methods to the ACTN1-related genes to select prognostic factors. *In vitro* experiments confirmed the pro-resistance and pro-metastatic functions of ACTN1 in osimertinib-resistant LC cells.

**Conclusion:**

ACTN1 was discovered to induce malignant progression and formation of EGFR-TKI resistance. Targeting ACTN1-related pathways may provide novel insights to treat LCBM and overcome intracranial EGFR-TKI resistance.

## 1 Introduction

Lung cancer (LC) remains the leading cause of cancer-related mortality and ranks as the second most frequently diagnosed cancer category across both genders globally, as highlighted by the statistics reported in the Global Cancer Statistics 2020 (Sung et al., 2021). Currently, conventional treatment modalities including surgical resection, radiotherapy, chemotherapy, targeted therapy and immunotherapy have yielded satisfactory outcomes in a subset of patients, thereby improving the overall survival rates and quality of life (Lahiri et al., 2023; Reck et al., 2022). Nonetheless, the interpatient heterogeneity, stemming from environmental risk factors and varied genetic profiles, frequently results in treatment failure and recurrence, posing a persistent threat to patients’ lives (Li et al., 2023; Meyer et al., 2024). Consequently, it is imperative to identify novel biomarkers to formulate personalized treatment strategies, reduce the emergence of drug resistance and ensure the therapeutic efficacy.

Lung cancer brain metastasis (LCBM), recognized as a frequent complication of LC, accounts for 50% of the incidence in patients with brain metastatic disease and poses a significant clinical challenge to further prognosis (Yousefi et al., 2017). A systematic review has underscored the deleterious impacts of LCBM, encompassing diminished quality of life, shortened survival, and a substantial economic burden for the majority of patients, reinforcing the critical need to elucidate the underlying mechanisms of metastasis and drug resistance (Peters et al., 2016). Several hypotheses have been proposed to explain the possible mechanisms underlying LCBM. Blood-brain barrier (BBB), a selectively permeable membrane, separates brain parenchyma from the systemic circulation and effectively protects the central nervous system from external invasion (Hajal et al., 2021). The BBB is composed primarily of endothelial cells, astrocytes and pericytes, all of which undergo pathological alterations in the early stages of LCBM (Wu et al., 2025a; Er et al., 2018). Firstly, circulating tumor cells can adhere to the endothelium of cerebral blood vessels to destroy the pre-existing intercellular tight junctions, and thereby facilitate transmigration and metastatic colonization in the brain (Zheng et al., 2024; Xia et al., 2024). Moreover, the underlying crosstalk between activated astrocytes and tumor cells has been identified as a key mechanism to drive metastasis. Evidence suggests that tumor cells can initially recruit and activate astrocytes through releasing interleukins and other inducing factors. Simultaneously, the matrix metalloproteinases derived from astrocytes further disrupt BBB integrity and secreted cytokines can promote LCBM progression (Wu D. et al., 2021; Qu et al., 2023; Tang et al., 2024; Priego et al., 2025).

Except for the existence of BBB, metabolic reprogramming has also been revealed as a regulatory process for mediating brain metastasis (BM). For instance, alterations in glutamine metabolic pathway have been reported to influence the GABA production, thereby fostering an immunosuppressive tumor microenvironment (TME) and inducing the development of LCBM (Xie et al., 2025; Chen et al., 2025). Therefore, targeting dysregulated metabolic pathways may provide novel opportunities to prevent the progression of LCBM.

Currently, the advent of single-cell technique compensates for the limitations characterized by conventional bulk transcriptome techniques by enabling the detection of rare cell populations (Liang and Fu, 2017; Olbrecht et al., 2021; Zhou et al., 2020). Previous studies have illustrated the relatively immunosuppressive TME in LCBM compared to primary lung tumors and other brain malignancies, which may facilitate metastasis and colonization of LC cells (Lah et al., 2020; Sun et al., 2022). The extracellular matrix and blood-derived immune cells constitute major components of TME, and their interaction with metastatic LC cells play a critical role in the initiation and progression of BM (Wang et al., 2022; Wang X. et al., 2024; Kim et al., 2025). Although multiple lines of evidence support the possible mechanism of BM, the exact intrinsic regulatory pathways and actionable therapeutic targets remain to be discovered.

Given the challenges posed by metastasis, numerous studies have deciphered that resistance to epidermal growth factor receptor-tyrosine kinase inhibitors (EGFR-TKIs), a cornerstone of targeted therapy for LC, often develops in patients with LCBM (Liu et al., 2021; Chiu et al., 2022; Hyun et al., 2020). In the era of targeted therapy, third-generation EGFR-TKIs, represented by Osimertinib, have been widely adopted as the first-line treatment of advanced LC patients and achieved remarkable therapeutic effects. Unfortunately, acquired resistance remains a major limitation to their long-term efficacy. According to the prior studies, resistant mechanisms are categorized into two categories: EGFR-dependent and EGFR-independent mechanisms. EGFR-dependent mechanisms mainly include mutations such as C797, C792, G796 and G724, whereas EGFR-independent mechanisms consist of MET amplification, MET mutations, HER2 alterations and aberrant activation of other bypassing pathways (Dong et al., 2021; Chmielecki et al., 2023; He et al., 2021). However, In the context of BM, alterations of specific proteins may serve as the main cause of resistance (Liu et al., 2021; Wu et al., 2025b), but the exact mechanisms have not been elucidated yet. Therefore, clarifying the correlation between LCBM and the accompanying phenomenon of EGFR-TKI resistance may help reverse the resistance within the intracranial environment and improve the therapeutic efficacy of EGFR-TKIs.

Thus, in the present study, we explored the capacity of alpha-actinin1 (ACTN1) in promoting drug resistance and metastasis of LC cells through integrating single-cell and bulk RNA sequencing data. In mammal, ACTN is classified into four subtypes: ACTN1, ACTN2, ACTN3 and ACTN4 (Murphy and Young, 2015; Foley and Young, 2014). As a cytoskeletal protein, ACTN1 serves as a key mediator in multiple muscle and non-muscle functions, encompassing cell-matrix adhesion, focal adhesion, integrin binding and cell migration. Previous studies have manifested that cytoskeletal-associated proteins not only play a crucial role in the cytoskeleton assembly, but also facilitate the formation of mitotic spindle in cancer cells, suggesting their potential as biomarkers for forecasting drug-resistance, cancer initiation and metastatic progression (Klingler-Hoffmann et al., 2019; Ayanlaja et al., 2021). Moreover, the tumor-promoting and drug-resistance properties have been identified in other cancer types, including hepatocellular carcinoma, ovarian cancer and breast cancer, as well (Cao et al., 2020; Chen et al., 2021; Gu et al., 2023).

In this study, with the aid of machine learning techniques and functional enrichment analyses, we have elucidated the underlying signaling pathways and validated the prognostic value of the ACTN1-related model. Leveraging cellular assays, we have confirmed the biological function of ACTN1 in driving malignant phenotypes and contributing to drug resistance in LC.

## 2 Materials and methods

### 2.1 Data acquisition and preprocessing

The scRNA-seq data from 7 LCBM lesions data were retrieved from the Gene Expression Omnibus (GEO) database (http://ncbi.nlm.nih.gov/geo/). Specifically, datasets GSE234832 (Song et al., 2023), GSE143423 (Wang et al., 2025) and GSE186344 (Gonzalez et al., 2022) were utilized to construct a comprehensive single-cell atlas of the LCBM microenvironment. Detailed information for all datasets is provided in Table 1. Samples derived from other cancer types were removed, and the remaining data were retained for their high sequencing quality and relevance to LCBM.

**TABLE 1:** Summary of scRNA-seq datasets from GEO database used to construct an integrated single-cell atlas of LCBM lesions (n = 7).

Dataset accession	Original studies	Sample ID	Tissue
GSE234832	Single-cell sequencing reveals the landscape of the human brain metastatic microenvironment	GSM7475327 GSM7475328	Lung cancer brain metastasis
GSE143423	Single-cell map of diverse immune phenotypes in the metastatic brain tumor microenvironment of nonsmall-cell lung cancer	GSM4259354 GSM4259355 GSM4259356	Lung cancer brain metastasis
GSE186344	Cellular architecture of human brain metastases	GSM5645895 GSM5645896	Lung cancer brain metastasis

Moreover, bulk RNA-seq data from 491 LC samples across distinct stages, along with corresponding clinical information, were obtained from the TCGA-LUAD dataset via the UCSC Xena platform (http://xena.ucsc.edu/). Affymetrix microarray datasets, serving as validation transcriptome cohorts, were sourced from GSE30219 (Rouss et al., 2013), GSE31210 (Okayama et al., 2012), and GSE72094 (Schabath et al., 2016). To achieve favorable comparability across different datasets, we transformed the raw Transcripts Per Million (TPM) values to log_2_(TPM+1). This transformation aids in stabilizing the variance and improving the approximate normality of the data distribution. Additionally, to mitigate potential batch effects across datasets, the “sva” package was applied. This package uses an empirical Bayes framework to effectively identify and remove batch-associated variability, thereby enhancing the consistency and reliability of the integrated dataset.

To validate transcriptomic findings at the protein level, immunohistochemistry (IHC) staining images were systematically retrieved from the Human Protein Atlas (HPA) database (https://www.proteinatlas.org/) to examine the protein expression patterns of candidate genes. Additionally, we obtained spatial transcriptomic (ST) data from osimertinib-resistant LC samples in GSE267960 datasets and selected four primary LC tissues as controls from the E-MTAB-13530 dataset in the ArrayExpress database (https://www.ebi.ac.uk/biostudies/arrayexpress). A summary of the ST datasets used is provided in Table 2.

**TABLE 2:** Spatial transcriptomics dataset of primary and osimertinib-resistant lung cancer tissues.

Dataset accession	Original studies	Sample ID	Tissue
GSE267960	VEGF Signal Complexity Confers Resistance to Atezolizumab, Bevacizumab, Carboplatin, and Paclitaxel in EGFR-Tyrosine Kinase Inhibitor-Resistant Non-Small Cell Lung Cancer	GSM8282531 GSM8282532	Osimertinib-resistant lung cancer tissue
E-MTAB-13530	Single-cell and spatial transcriptomics analysis of non-small cell lung cancer	P10 P15 P16 P24	Primary lung cancer tissue

### 2.2 Processing of scRNA-seq data and cell type annotation

To delineate the comprehensive atlas of TME, we utilized the Seurat package (Zhao et al., 2024) for the integration and processing of LCBM samples. Initially, cells with inferior quality were excluded based on stringent criteria: (1) cells were required to contain 200–5,000 detected genes; (2) cells were required to have <5% mitochondrion-derived Unique Molecular Identifiers (UMIs). This filtering step ensured the retention of high-quality cells essential for accurate downstream analyses.

Subsequently, the data were normalized and log-transformed. The top 2,000 highly variable genes were identified using “FindVariableFeatures”. The normalized data were then scaled and underwent dimensionality reduction through the application of “ScaleData” and “RunPCA” functions. This step was crucial for capturing the most significant sources of variation across datasets. To reduce batch effects arising from the integration of distinct datasets, we employed the “Harmony” R package based on the top 15 PCA components. The final processed data were visualized by Uniform Manifold Approximation and Projection (UMAP) (Do and Canzar, 2021), a technique renowned for its ability to accurately represent the underlying data structure and effectively display cell characteristics in a low-dimensional space. For cell clustering, we employed “FindNeighbors()” and “FindClusters()” functions and ultimately generated 19 clusters with the resolution set to 0.5.

Based on published studies by Tagore et al. (2025), Wang et al. (2023) and Fu et al. (2024) et al., together with cell markers from the CellMarker database (http://bio-bigdata.hrbmu.edu.cn/CellMarker/), all clusters were manually annotated as B cells, endothelial cells, epithelial cells, fibroblasts, myeloid cells, neural stem cells (NSCs), oligodendrocytes and T cells. This annotation process involved comparing the gene expression profiles of each cluster with known markers and characteristics of various cell types to ensure accurate and reliable classification.

### 2.3 Scissor analysis and drug sensitivity analysis

To evaluate the perturbations induced by multiple drugs across cell clusters, we used “Beyondcell” R package (Fustero-Torre et al., 2021) to infer the susceptibility of different cell subpopulations to candidate drug treatments. The analysis involved employing an algorithm that incorporates two key feature sets: the drug perturbation feature set (PSc) and the drug sensitivity feature set (SSc). Leveraging this algorithm, we calculated the Beyondcell score (BCS), which quantifies the predicted transcriptional response to different drugs. The switch point (SP), which is a quantitative measure of drug response homogeneity, was also computed to reflect cell-to-cell variability within each subpopulation. Joint interpretation of BCS and SP enabled us to assess the influence of drug-induced transcriptional changes on cellular responses.

The Scissor algorithm can identify the cell clusters that are significantly correlated with the resistant phenotype, which were annotated as Scissor^+^ cells. Conversely, the negatively related cell clusters were designated as Scissors^−^ cells. To quantify the degree of sensitivity in the TCGA-LUAD dataset, we utilized the single-sample Gene Set Enrichment Analysis (ssGSEA) algorithm to calculate enrichment scores. These scores provided a quantitative measure of the presence and activity of gene sets associated with EGFR-TKI sensitivity or resistance within each sample. By integrating the processed single-cell and bulk transcriptome sequencing data, we extracted the relatively resistant cell clusters, which were marked as Scissor^+^ for subsequent investigation.

### 2.4 Identifying biological characteristics of scissor^+^ epithelial cells via high-dimensional weighted gene co-expression network analysis (hdWGCNA)

HdWGCNA was employed to elucidate the data structure of the projected components, facilitating the identification of biologically relevant functions or phenotypes by constructing gene co-expression networks (Morabito et al., 2021; Morabito et al., 2023). At the single-cell level, we utilized the R package “hdWGCNA” to construct a scale-free topology model. The construction process involved selecting an appropriate soft-thresholding power to ensure optimal connectivity within the network. Soft thresholds were determined using the “TestSoftPowers” function, and the selected soft-thresholding power was crucial for balancing the resolution and robustness of the gene co-expression network. Genes were clustered into different modules using weighted gene co-expression network analysis (WGCNA) (Guo et al., 2023). Each module represents a set of genes with highly correlated expression patterns, indicative of shared biological functions or regulatory mechanisms.

### 2.5 Pseudotime analysis and cell-cell communication analysis

Pseudotime trajectory analysis is a powerful tool for reconstructing the temporal dynamics of cellular differentiation and development. This approach arranges individual cells according to their gene expression profiles along a putative chronological axis, thereby partitioning samples into multiple cell populations at various stages of differentiation. The resulting lineage dendrogram provides an intuitive visualization of the predicted differentiation and developmental trajectories of cells (Wang et al., 2023). Trajectory analysis was conducted using the “Monocle2” package to construct a differentiation trajectory of epithelial cells. The “DDRTree” function was applied for dimensionality reduction, employing default settings to ensure robust and accurate trajectory inference. The pseudotime trajectory was visualized using the “plot_cell_trajectory” function, which generates a clear and interpretable representation of the cellular differentiation process.

To elucidate the intercellular communication networks within the TME, we performed cell-cell communication analysis using the “CellChat” R package (Jin et al., 2021). This package facilitates the identification and quantification of ligand-receptor (LR) interactions among different cell types, providing insights into the signaling pathways that mediate cellular communication. By analyzing these interactions, we aimed to uncover regulatory mechanisms that influence cellular behavior and contribute to the maintenance of the TME.

### 2.6 Identification of the differentially expressed genes (DEGs) and gene enrichment analysis

The DEGs between the high- and low-risk groups in TCGA-LUAD were determined using the “DESeq2” package (Love et al., 2014), with the thresholds of |log_2_foldChange| ≥ 1 and adjusted *p*-value <0.05. The potential functions of LCBM signature were assessed by Gene Ontology (GO) enrichment analysis, Kyoto Encyclopedia of Genes and Genomes (KEGG) and hallmark pathway enrichment analysis using the “clusterProfiler” R package (Wu T. et al., 2021). Changes in biological function over pseudotime were visualized utilizing the “ClusterGVis” and “org.Hs.eg.db” R packages (Ma et al., 2024). To define the drug-response landscape of LCBM, we identified genes that induce the EGFR-TKI resistance by conducting differentially expressed analysis on the GSE231938 and GSE262528 datasets.

### 2.7 Identification of risk factors and construction of a nomogram

Univariate and multivariate Cox regression analyses were employed to evaluate the prognostic value of ACTN1 expression. A nomogram was generated to predict the OS of patients by conducting “rms” and “replot” R packages (Wang H. et al., 2024). The nomogram integrates ACTN1 expression levels with relevant clinicopathological parameters, providing a user-friendly graphical tool for individualized risk assessment. The construction of calibration curves and decision curve analysis (DCA) assessed the predictive performance of the nomogram. Calibration curves evaluated the agreement between predicted and observed survival probabilities, whereas DCA quantified the clinical net benefit of using the nomogram for decision-making. These validation steps ensured the reliability and practical utility of the nomogram in clinical settings.

### 2.8 Immune infiltration analysis

To explore the immune landscape of the TME, we applied six algorithms (MCPcounter, quantiseq, xCELL, TIMER, Estimate and EPIC) to quantify the proportion of various immune cell populations using the “IOBR” package (Zeng et al., 2021). This comprehensive approach allowed for a multifaceted assessment of the immune contexture. In addition to the algorithm-based quantification, we calculated the abundance of 21 immune cell types using the ssGSEA algorithm. This method provided a complementary and robust validation of immune cell composition, thereby ensuring the reliability of our findings. Due to the intra-tumoral heterogeneity and the variable efficacy of immunotherapy in advanced LC patients, we investigated the expression profiles of immune checkpoints across distinct risk subgroups. By integrating these immune infiltration analyses, we aimed to provide a comprehensive understanding of the immune landscape within the TME, and highlight potential biomarkers and therapeutic targets for improving the survival outcomes in relevant patients.

### 2.9 Establishing a prognostic signature via integrative 101 machine learning approaches

To reduce the risk of overfitting, we utilized 10 algorithms encompassing random survival forest (RSF), elastic network (Enet), Lasso, Ridge, stepwise Cox, CoxBoost, partial least squares regression for Cox (plsRcox), supervised principal components (SuperPC), generalized boosted regression modelling (GBM), and survival support vector machine (survival-SVM), to perform hybrid computing and construct a highly accurate prognostic model. Specifically, TCGA-LUAD served as the training cohort for screening prognostic genes associated with LCBM, whereas GSE31210, GSE30219 and GSE72094 datasets were employed as validation cohorts to check the accuracy of the prognostic model. On the basis of leave-one-out cross-validation (LOOCV) framework, the 101 combinations derived from 10 algorithms were applied to narrow down the candidate prognostic genes (Liu et al., 2022).

To evaluate the predictive performance of all models and select the most optimal one, we first calculated Harrell’s concordance index (C-index). The C-index can reflect the discriminative ability between predictive and observed outcomes, and it is applicable to censored survival data, making it suitable for comparing models across cohorts with different follow-up durations (Poirion et al., 2021; Clift et al., 2023; Luo et al., 2025). Therefore, it is reasonable to use C-index as a weighted metric for model evaluation. Moreover, we employed the mean area under the curve (AUC) value at 1, 3 and 5 years for each model to assess the accuracy, sensitivity and specificity (Huang et al., 2022; Huang et al., 2025). Kaplan-Meier (KM) curves were then constructed with the aid of “survival” and “survminer” R packages to delineate the survival differences. Both the time-dependent AUC value and KM analysis serve as complementary measures to assess performance.

### 2.10 Analysis of ST data

ST data from primary and osimertinib-resistant tissues were initially normalized and scaled utilizing the “SCTransform” function for downstream analysis (Nesari et al., 2026). Subsequently, we extracted highly variable features, and the data underwent dimensionality reduction via the “RunPCA” function. To mitigate the batch effect originating from different datasets, we employed the “RunHarmony” function using the first 20 principal components (PCs). Ultimately, we identified and visualized ACTN1 expression across samples using the “SpatialFeaturePlot” function.

### 2.11 Visual knockout assay

To evaluate the biological role of ACTN1 in epithelial subclusters, we initially performed a visual knockout (KO) assay. After extracting epithelial cells, we applied the “scTenifoldKnk” R package (Osorio et al., 2022), a workflow that enables *in silico* gene knockdown based on scRNA-seq data. Using a threshold of *p*-value <0.05 together with fold change criteria, we identified differentially perturbed genes for further functional enrichment analysis.

### 2.12 Cell culture and generation of an osimertinib-resistant cell line

The human NSCLC cell line PC9 was sourced from the Shanghai Academy of Sciences (Shanghai, China). Upon receipt, cells were cultured in RPMI-1640 medium supplemented with 10% fetal bovine serum (FBS) and 1% penicillin-streptomycin (P/S). and they were maintained at 37 °C in a humidified incubator with 5% CO_2_.

The osimertinib-resistant PC9 cell line was previously constructed in our laboratory using a dose-escalation strategy. Briefly, parental PC9 cells in the logarithmic growth phase were exposed to gradually increasing concentration of osimertinib (#HY-15772, MedChemExpress, China). Treatment was initiated at 10 nmol/L, and the concentration was incrementally increased by 10–20 nmol/L per step, until a stable final concentration of 3000 nmol/L was reached. The entire induction of osimertinib resistance was completed over approximately 30 weeks.

### 2.13 Quantitative real-time PCR (qPCR)

Total RNA was extracted from PC9 and osimertinib-resistant PC9 cells using TRI reagent (Biosharp, China) according to the manufacturer’s protocol. First-strand cDNA was synthesized using the Hieff NGS® first cDNA Synthesis Kit (#12946ES96, YEASEN, China) following the manufacturer’s instructions. qPCR was performed using SYBR Green qPCR Master Mix (#BL1565A, Biosharp, China) under the following cycling conditions: initial denaturation at 95 °C for 15 s, followed by 40 cycles of 95 °C for 15 s and 60 °C for 30 s. Relative gene expression was normalized to GAPDH and subsequently calculated using the 2^(-ΔΔCt)^ method. Primer sequences used for qPCR are listed below:

ACTN1-Forward (HUMAN): CGC​CTC​TTT​CAA​CCA​CTT​TG, ACTN1-Reverse (HUMAN): TCA​TGA​TTC​GGG​CAA​ACT​CT; GAPDH-Forward (HUMAN): GGA​GCG​AGA​TCC​CTC​CAA​AAT, GAPDH-Reverse (HUMAN): GGC​TGT​TGT​CAT​ACT​TCT​CAT​GG.

### 2.14 Cell transfection and western blotting

For the cell transfection assay, cells were serum-starved for 20–24 h and then seeded into 6-well plates at an appropriate transfection density (5.0 × 10^4^cells/well). A transfection mixture containing siRNA, jetPRIME® transfection reagent (Ployplus, France) and culture medium was prepared in advance, and subsequently added into cells at the logarithmic growth phase. After 6-h incubation, we harvested transfected cells for further analysis. Constructed siRNA sequences were as follows:

ACTN1-siRNA: GGA​CAC​AGA​UCG​AGA​ACA​UTT
Control-siRNA: UUC​UCC​GAA​CGU​GUC​ACG​UTT

To validate the silencing efficacy, we conducted Western blotting assay in siRNA-silenced and negative control (NC) cells. Extraction of protein was performed using RIPA lysis buffer (#BL504A, Biosharp, China) supplemented with protease and phosphatase inhibitors. The lysis assay was conducted on ice for 30 min and we extracted cytoplasmic proteins from the lysed solution by centrifugation at 12,000 rpm and 4 °C for 10 min. BCA protein assay kit (#EC0001, SparkJade, China) was utilized to detect protein concentration. Equal amounts of targeted proteins (20–40 μg) were separated by performing sodium dodecyl sulfate-polyacrylamide gel electrophoresis (SDS-PAGE) and proteins were transferred onto polyvinylidene fluoride (PVDF) membranes (EpiZyme, China) at a constant current of 220 mA for 1.5 h. Ultimately, the membranes were blocked with protein free rapid blocking buffer (1×) (EpiZyme, China) in Tris-buffered saline (#G0001-2L, Servicebio, China) with 0.1% Tween-20 (TBST) for 1 h at room temperature to prevent non-specific binding.

Primary antibodies used for incubation include anti-ACTN1 (#11313-2-AP) and anti-GAPDH (#60004-1-Ig) bought from Proteintech (Wuhan, China). After incubation with the appropriate horseradish peroxidase (HRP)-conjugated secondary antibody for 1–2 h at room temperature, protein bands were visualized using enhanced chemiluminescence (ECL) reagent (#BL520A, Biosharp, China) according to the manufacturer’s instructions. Images of the blots were acquired and recorded by the Chemi Dog 5200T automatic luminescence imaging system (Tanon, China). Protein band intensities were quantified using ImageJ software, and all experiments were repeated at least three times.

### 2.15 Cell viability assay

To evaluate the proliferation capacity of PC9-OR cells before and after gene silencing, we collected transfected cells and seeded them into 96-well plates at the concentration of 800 cells/well. After 8-h incubation to allow complete adhesion to the well, the original culture medium in each well was replaced with fresh medium containing 10% enhanced Cell Counting Kit-8 (CCK-8) reagent (#C0042, Beyotime, China). Experimental plates were incubated for 2 h, and the optical density (OD) values at 450 nm were measured using a microplate reader. The measurement processes were tested in triplicate, and cell viability in the NC and gene-silencing groups was assessed every 24 h for five consecutive days.

To performed drug sensitivity analysis, we then seeded parental and osimertinib-resistant PC9 cells into 96-well plates and maintained the cell concentration at 2,000 cells per well. Cells were subsequently incubated with the designated concentration of osimertinib and the cell proliferation capacity was evaluated with enhanced CCK-8 reagent, according to manufacturer’s instructions. Cell viability assays were independently repeated at least three times. The half maximal inhibitory concentration (IC_50_) value for each cell line was calculated using GraphPad Prism software (version 10.6).

### 2.16 Transwell assay

To assess the alterations in migration capacity, we performed Transwell assay after the transfection experiment. First, cells received serum-starved treatment for 24 h prior to the assay. Osimertinib-resistant and transfected PC9 cells were counted and 3.0 × 10^4^ cells suspended in 200 μL medium were seeded into the upper chamber of Transwell inserts (#14311, LABSELECT, China). While 600 μL culture medium with 1% FBS was placed in the lower chamber, serving as a chemoattractant. After 20 h of incubation at 37 °C, we fixed the migrated cells within lower chamber with 4% paraformaldehyde and stained them with 2.5% crystal violet for 30 min. Non-migrated cells on the upper surface were gently wiped off using damp cotton swabs for accurate observation. Cell numbers were captured under a 10× microscope and counted manually. All experiments were repeated at least three times.

### 2.17 Statistical analysis

Rationally, we employed R software (version 4.3.0) for the statistical analyses of single-cell and transcriptome sequencing data, whereas GraphPad Prism software (version 10.6) was used to quantitatively analyze and visualize experimental data. Pearson’s correlation test was applied to evaluate correlations between gene modules. For comparisons of immune cell fractions and immune checkpoint expression between groups with distinct ACTN1 expression levels, an independent t-test was applied when parametric assumptions were met. If these assumptions were violated, the non-parametric Wilcoxon rank-sum test was used as an alternative. All reported *p*-values were two-tailed, and a threshold of *p*-value <0.05 was considered statistically significant.

## 3 Results

### 3.1 ScRNA-seq unveiling the cellular compositions of LCBM and their responses to EGFR-TKIs

**FIGURE 1:**
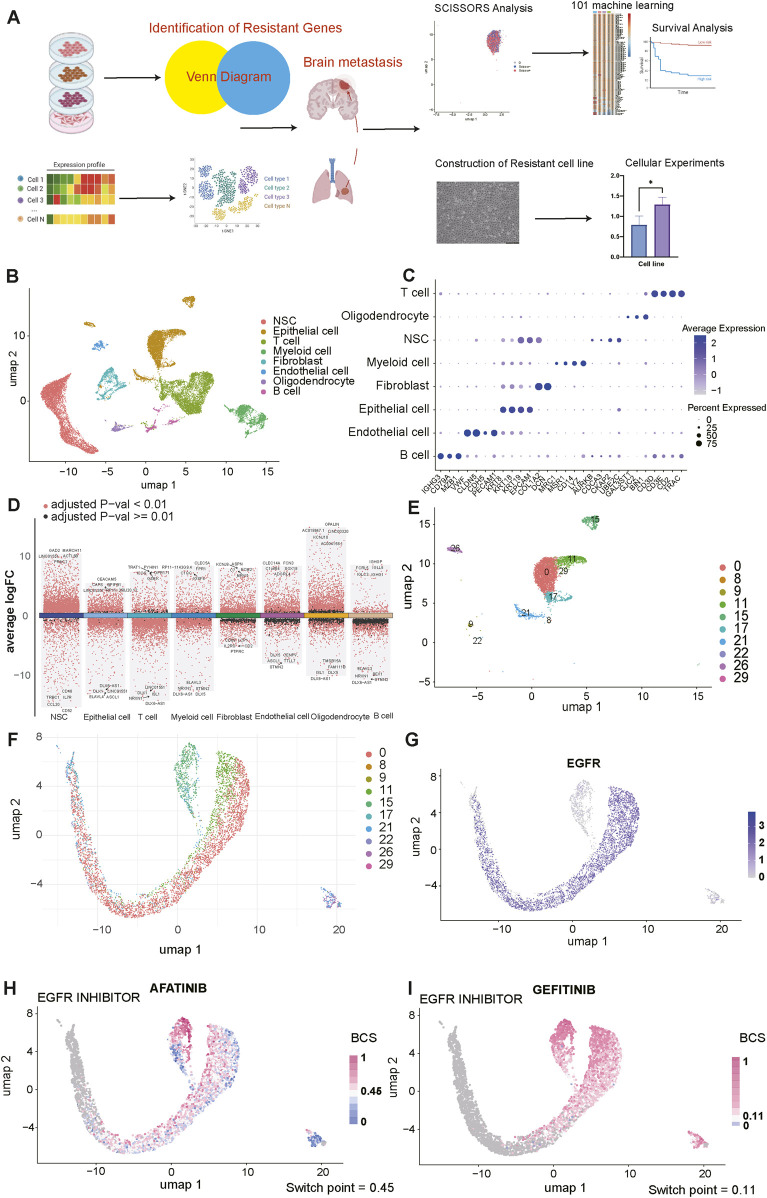
Integrating single-cell RNA sequencing (scRNA-seq) data to identify drug sensitivity heterogeneity of epithelial cells in the single-cell landscape of lung cancer brain metastasis (LCBM). **(A)** A flow chart of entire analysis designed in our study. Some pictures are supported by BioRender application. **(B)** Uniform manifold approximation and projection (UMAP) visualizations show the dimensionality reduction of scRNA-seq data and highlight the distribution of various cell types, including neural stem cells (NSCs), epithelial cells, T cells, myeloid cells, fibroblasts, endothelial cells, oligodendrocytes, and B cells. **(C)** The average expression levels of marker genes across different cell types, visualized by a scatter plot. **(D)** A volcano plot visualizing the results of differential expression analysis comparing diverse cell types. Genes are color-coded based on their expression levels, with red dots indicating significant differential expression (adjusted *p*-value <0.01) and black dots representing non-significant changes (adjusted *p*-value ≥0.01). **(E,F)** UMAP plots visualize the distribution of epithelial cells at high resolution (resolution = 0.8). **(G)** The expression landscape of epidermal growth factor receptor (EGFR) in epithelial cell clusters, calculated by the “Beyondcell” algorithm. **(H,I)** UMAP plots illustrate the distribution of epithelial cells in response to the EGFR inhibitors: afatinib and gefitinib. The color gradients from blue to red indicate lower to higher drug sensitivity, separated by the calculated switch point.

A schematic diagram in Figure 1A outlines the comprehensive workflow of our experimental and data analytical processes. To characterize the complex microenvironment of LCBM and uncover the potential mechanisms behind metastasis and drug resistance, we selected 7 representative LCBM tissue samples from three datasets in GEO database (GSE143423, GSE186344 and GSE234832) to generate and analyze scRNA-seq profiles. Baseline clinical and sample information of all samples is comprehensively documented in the corresponding original articles (Song et al., 2023; Wang et al., 2025; Gonzalez et al., 2022). After rigorous filtering procedures, which included constraining the proportion of cellular signatures and diminishing cells with high concentrations of mitochondrial and ribosomal genes, we retained a total of 22,157 LCBM cells and 29,432 genes for subsequent analysis. A dimensionality reduction methodology, UMAP, was then implemented to culminate in the categorization of 19 distinct major clusters. Based on conventional cell markers, all clusters were annotated as follows: B cells (IGHG3, CD79A, MZB1), endothelial cells (VWF, CLDN5, CDH5, PECAM1), epithelial cells (KRT8, KRT18, KRT19, EPCAM), fibroblasts (COL1A2, DCN), myeloid cells (MRC1, MSR1, CD14, LYZ), NSCs (AURKB, CDCA3, CKAP2, UBE2C), oligodendrocytes (GAL3ST1, GJC2, BIN1), T cells (CD3D, CD3E, CD2, TRAC) (Figure 1B). A bubble plot depicted the expression patterns of marker genes across distinct cell types within the LCBM tissues (Figure 1C). Additionally, DEGs were identified in each cluster, highlighting the distribution discrepancies, as visualized in a volcano plot (Figure 1D). Notably, epithelial cells constituted a significant proportion of all clusters, meriting further investigation, due to their potential role in targeted therapy resistance. In this regard, we isolated epithelial cells, which could be further subdivided into 10 distinct subclusters. We generated UMAP embeddings to visualize scRNA-seq changes induced by diverse agents, utilizing databases built within the Beyondcell platform (Figures 1E,F). Furthermore, we comprehensively evaluated the expression of EGFR across all clusters and assessed their susceptibility to commonly used EGFR-TKIs. Intriguingly, by calculating switch points, we observed a notable phenomenon within certain cell communities, particularly in cluster 0 (C0). Despite the homogeneous expression of EGFR across all clusters, epithelial cells within C0 exhibited distinct responsiveness to afatinib and gefitinib (Figures 1G–I). This observation prompted us to hypothesize that inherent epithelial-cell heterogeneity may be closely correlated with the emergence of resistance to EGFR-TKIs. We proposed that identifying genes associated with metastasis and drug resistance may potentially lay a foundation for reversing phenotypic transition and overcoming EGFR-TKI resistance in LCBM patients.

### 3.2 Identification of ACTN1 as a hub gene involved in the EGFR-TKI resistance based on scissors and hdWGCNA algorithms

**FIGURE 2:**
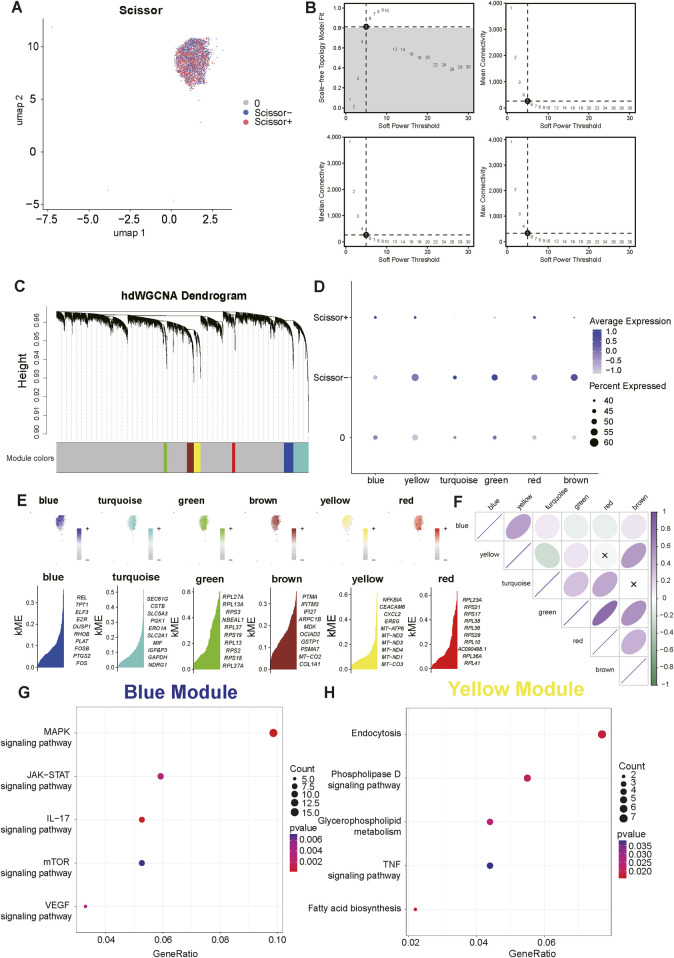
Exploring hub genes correlated with subpopulations of EGFR-TKI-resistant epithelial cells via high-dimensional Weighted Gene Co-expression Network Analysis (hdWGCNA) analysis. **(A)** UMAP visualizations of scRNA-seq data highlight epithelial cell populations in different Scissor groups. Scissor^+^ epithelial cells exhibit features of EGFR-TKI resistance. **(B)** Plots present a comprehensive analysis of network metrics, including scale-free topology model fit, mean connectivity, median connectivity, and maximum connectivity, as functions of different soft-thresholding powers. **(C)** A hierarchical clustering dendrogram from the hdWGCNA analysis exhibits the hierarchical relationships among the identified modules. **(D)** A scatter plot visualizes the association between different modules and Scissor identity. **(E)** This heatmap presents a comparative analysis of gene expression data across six distinct modules. The top panel shows the enrichment atlas of each module in UMAP plots. The bottom panel provides a ranked list of the top ten genes based on their kME values within each module. **(F)** A heatmap visualizes the correlation matrix for each module. **(G,H)** Scatter plots display the Kyoto Encyclopedia of Genes and Genomes (KEGG) enrichment results of hub genes in the blue and yellow modules.

To establish a robust and broadly applicable set of genes associated with EGFR-TKI resistance, we systematically identified genes that were markedly upregulated in LC cells exhibiting established resistant phenotypes by acquiring pre- and post-resistant samples from two GEO datasets (GSE231938 and GSE262582). Consequently, 487 concurrently upregulated resistant genes were selected for further analysis (Supplementary Figure S1). We subsequently employed the ssGSEA algorithm to investigate the resistance landscape in 491 TCGA-LUAD samples and the results enabled us to decipher high- and low-resistance groups based on the median calculated score as a threshold. To specifically interrogate LCBM cells with EGFR-TKI resistance, we utilized Scissors algorithm. By leveraging TCGA-LUAD samples and scRNA-seq data from C0, as previously described, we discerned 987 Scissors^+^ LCBM cells that exhibited a resistance-associated transcriptional phenotype (Figure 2A).

Furthermore, we conducted hdWGCNA to characterize co-expression programs within the Scissors^+^ population. When the soft power threshold (β) reached five, the entire co-expression network approximated a scale-free distribution, and the model connectivity was satisfactory, providing a robust framework for pinpointing key molecular features associated with resistance (Figure 2B). Six dominant gene modules, designated as blue, yellow, turquoise, green, red and brown, were identified and clearly represented in a dendrogram (Figure 2C). Additionally, a bubble plot manifested a significant correlation between the Scissors algorithm results and these distinct modules, with the blue, yellow and red modules positively associated with Scissors^+^ epithelial cells (Figure 2D). The calculation of kME values, which represent the eigengene-based connectivity of various genes, facilitated the assessment of gene weights within different modules. Consequently, we focused on the top 10 genes ranked by kME in each module and visualized their overall distribution in a UMAP visualization (Figure 2E). The intensity of association between diverse modules was depicted in a bubble diagram (Figure 2F) and we obtained hub genes in blue and yellow modules, which exhibited significant inter-correlation, for subsequent analysis.

Leveraging KEGG enrichment analysis, we defined their biological functions of these hub genes from the resistance-associated modules: genes obtained from blue module were mainly enriched in MAPK, JAK-STAT, IL-17, mTOR signaling pathways, whereas those from yellow module were mainly associated with endocytosis, phospholipase D signaling pathway and glycerophospholipid metabolism (Figures 2G,H). Genes with high intramodular connectivity, as determined by kME values, were extracted and meticulously selected by conducting survival analysis (Supplementary Figure S2). This comprehensive process culminated in the identification of ACTN1 as a key candidate associated with the development of drug resistance and unfavorable survival outcomes.

### 3.3 Analysis of trajectory inference and intercellular communication of epithelial cells

**FIGURE 3:**
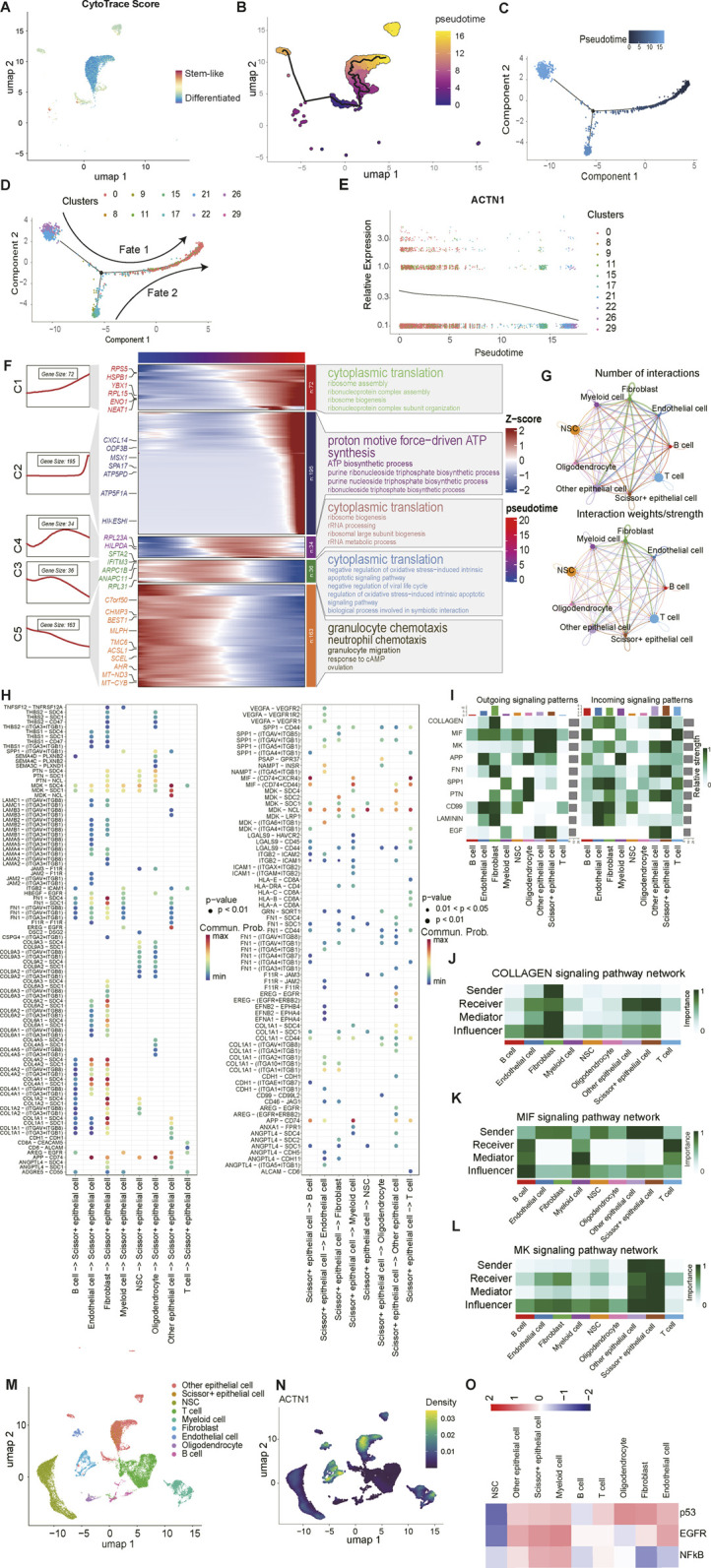
Cell trajectory inference and intercellular communication analysis of epithelial cells. **(A)** Based on the CytoTrace Score, the stem state of epithelial cells is indicated by colors. **(B)** A UMAP plot highlights the trajectory of cellular differentiation over pseudotime. **(C)** The color gradient indicates pseudotime, with cells color-coded based on timepoints. **(D)** A plot displays the distribution of different clusters, categorized into two developmental trajectories: cell fate 1 and cell fate 2. **(E)** A plot illustrates the dynamic expression of ACTN1 in all clusters over pseudotime. **(F)** A heatmap visualizes the expression dynamics of genes across different pseudotime stages of the biological process. Genes are grouped into clusters (C1-C5) based on their expression patterns and are enriched into the associated biological processes. **(G)** Network plots display the number and weights of interactions between different cell types. **(H)** Dot plots exhibit the interactions between Scissors^+^ epithelial cells and other cell types through different ligand-receptor (LR) pairs. **(I)** A heatmap illustrates relative strengths of signaling pathways in different cell types, focusing on both outgoing and incoming signaling patterns. **(J–L)** Heatmaps visualize the importance and role of various cell types within the computed COLLAGEN, MIF and MK signaling networks, respectively. **(M,N)** UMAP plots exhibit the enrichment of ACTN1 in the single-cell landscape. **(O)** A heatmap illustrates the activation of signaling pathways, including p53, EGFR, and NFκB, across different cell types.

To accurately trace the dynamic state transitions among epithelial cell clusters within the TME, we initially evaluated the differentiated status and simulated the chronological changes of cell communities, providing insights into the evolutionary trajectory of epithelial cell populations (Figures 3A–C). Through meticulous observation, we delineated two differentiated routes and detected C0 predominantly represented the late stage of cellular evolution, as illustrated by developmental analysis (Figure 3D). Besides, we noted that the relative expression pattern of ACTN1 change concomitantly with the shifts in cellular communities, with the highest expression levels observed in C0 (Figure 3E). Therefore, it is reasonable to designated the C0 as the ACTN1^+^ epithelial subcluster.

The altered genes and transformations in biological functions, particularly in term of biological processes, were visualized using heatmaps (Figure 3F). By analyzing the alteration of biological functions and developmental trajectories, we discovered a significant recruitment of granulocytes towards ACTN1^+^ epithelial subcluster. This migration can be attributed to chemokine secretion.

Additionally, we discovered aberrant signaling between epithelial cells and other cell types, by leveraging cell-cell communication analysis. Considering both frequency and strength of interactions, it is particularly noteworthy to emphasize the intrinsic connections between epithelial cells and other cell categories (Figure 3G). The potential interactions between ligands and receptors illustrated the complex crosstalk among different cell types, and bubble plots manifested the intensity of interactions involving epithelial cells and other cell clusters (Figure 3H). The results demonstrated that epithelial cells influenced other cells by activating MIF and MDK signaling pathways, notably through MIF-(CD74+CXCR4) and MIF-(CD74+CD44) interactions. Conversely, the proliferation of epithelial cells was impacted by fibroblasts primarily through COLLAGEN signaling pathways, encompassing COL1A1/COL1A2/COL4A2-SDC1/SDC4 interactions. Subsequently, we delineated and depicted the top 10 incoming and outgoing signals, which exerted significant influence on the remodeling of TME in a heatmap (Figure 3I). The color intensity reflected the strength of signals in specific cell types, with darker shades indicating a higher likelihood of biological significance. Similarly, the top three signaling pathways, including COLLAGEN, MIF and MK, warrant rigorous investigation to shed light on the actual roles of distinct cell communities in the formation of an anomalous TME. Focusing on the biological activities of epithelial cells, we observed that they primarily acted as receivers of COLLAGEN signals influenced and sent by fibroblasts (Figure 3J). Conversely, they functioned as a sender to regulate the MIF signaling pathway network (Figure 3K), suggesting their influence on immune responses and inflammation. Notably, epithelial cells were observed to participate in the entire cascade of the MK signaling pathways transmission, as evidenced by the heatmap (Figure 3L), underscoring their multifaceted role in cellular communication within the TME.

Ultimately, we defined the enrichment of ACTN1 within the single-cell landscape (Figures 3M,N) and observed upregulation of p53, EGFR and NFκB signaling pathways in ACTN1^+^ epithelial cells (Figure 3O). Recognizing these intercellular interactions is crucial for advancing the development of reliable targeted therapies that aimed at eliminating aberrant signals and restoring anti-tumor responses.

### 3.4 Survival analysis and functional investigation of different ACTN1 expression profiles

**FIGURE 4:**
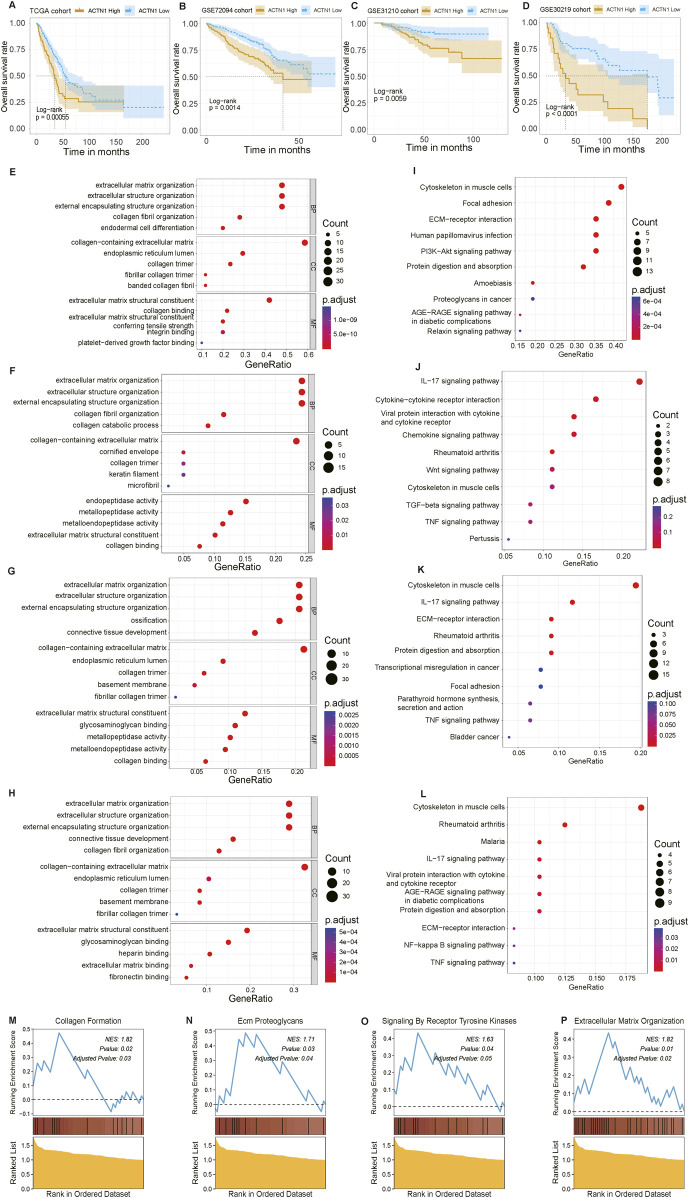
Delving into the survival differences and functional changes between high- and low-ACTN1 expression groups. **(A–D)** Kaplan-Meier (KM) analyses were conducted across multiple datasets (TCGA, GSE72094, GSE31210, GSE30219 cohorts), illustrating the relevant unfavorable prognosis in ACTN1-high group. **(E–H)** Gene Ontology (GO) analyses of differentially expressed genes (DEGs) in the two ACTN1 expression group. **(I–L)** KEGG analyses were conducted with DEGs. **(M–P)** Enriched biological pathways in patients with high ACTN1 expression, encompass collagen formation, ECM proteoglycans, signaling by receptor tyrosine kinases, and extracellular matrix organization.

To further clarify the influence of ACTN1 on patients’ survival, we conducted a comprehensive survival analysis across multiple datasets consisting of TCGA-LUAD, GSE72094, GSE31210 and GSE30219. Patients were stratified into high- and low-expression groups based on the optimal cutoff value for ACTN1 expression, and the group with ACTN1 expression exhibited significantly worse outcomes than the low expression group (P < 0.01) (Figures 4A–D). These findings indicate that ACTN1 is a promising therapeutic target.

Functional enrichment analyses were performed to reveal the potential biological functions of ACTN1-related genes. To explore this issue, we identified DEGs between the ACTN1-high and ACTN1-low groups and elucidated their roles by employing GO, KEGG enrichment analysis, and GSEA. Initially, GO analysis demonstrated that ACTN1-associated genes are similarly involved in the organization of the extracellular matrix (ECM), extracellular structure and external encapsulating structure within all datasets (Figures 4E–H). Moreover, the biological functions of genes implicated in cytoskeleton regulation, focal adhesion, ECM-receptor interaction, PI3K-Akt and IL-17 signaling pathway, were elucidated by KEGG analysis (Figures 4I–L). Based on these observations, we proposed that ACTN1 may regulate and activate both IL-17 and PI3K-Akt signaling pathways. This regulatory influence could modulate the development of ECM, thereby inducing the proliferation of LC tissues. GSEA results further underscored the activation of collagen generation-related pathways, including collagen formation, ECM proteoglycans and ECM organization, aligning with the elevated level of TKI-related signals (Figures 4M–P).

### 3.5 Establishment of ACTN1-related nomogram and immune characteristics of different expression groups

**FIGURE 5:**
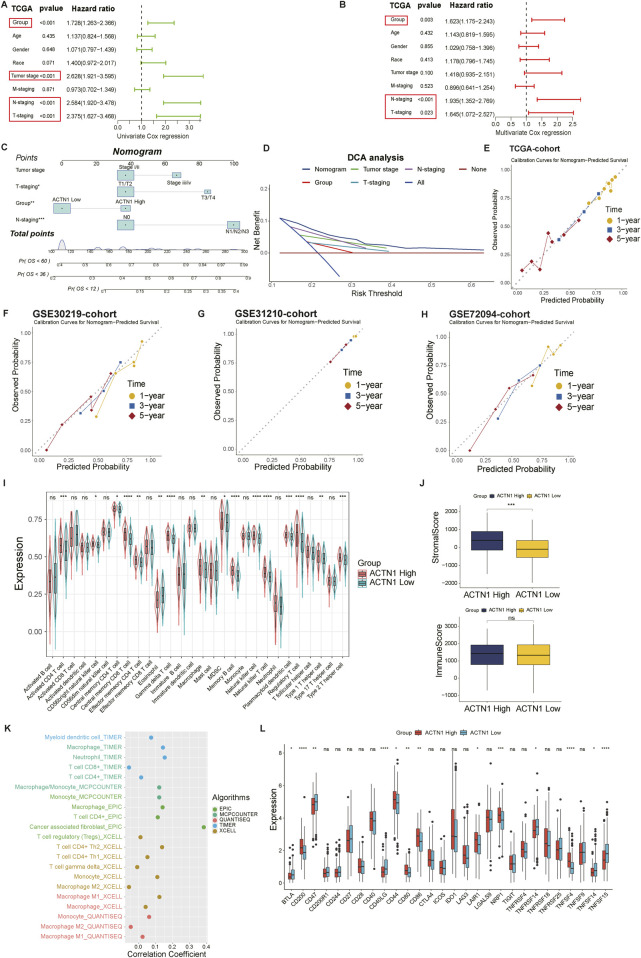
Establishment and validation of a nomogram, along with the investigation of immune profiles. **(A)** Univariate analysis of the clinicopathological parameters and ACTN1 expression group in the TCGA-LUAD cohort. **(B)** Multivariate analysis of the clinicopathological parameters and ACTN1 expression group in the TCGA-LUAD cohort. **(C)** A nomogram predicts 1-year, 3-year and 5-year overall survival (OS) of patients in TCGA cohort by integrating indicators (tumor stage, pathological T-staging, pathological N-staging and ACTN1 expression group). **(D)** Decision curve analysis (DCA) is conducted to assess the performance of the nomogram. **(E–H)** Calibration plots show the fitting degree between predicted and observed survival probability in the training and validation cohorts (TCGA, GSE30219, GSE31210 and GSE70294). **(I)** The immune infiltration profiles of 28 immune cell types in ACTN1-high and -low expression groups. **(J)** Stromal and immune scores are calculated and compared, as shown in these box plots. **(K)** The distribution landscape of immune cells is calculated through multiple algorithms. **(L)** The expression of immune checkpoints is shown in a box plot. (*p*-value ≥0.05, ns; *p*-value <0.05, *; *p*-value <0.01, **; *p*-value <0.001, ***; *p*-value <0.0001, ****).

To assess the prognostic implications of distinct clinicopathological parameters and ACTN1 expression, we conducted univariate Cox regression analysis. The results, depicted in a forest plot, clearly manifested a statistically significant association between poor outcomes and several factors, including ACTN1 expression group, tumor stage, pathological T-staging and pathological N-staging (all with hazard ratios (HRs) > 1, *P* < 0.001) (Figure 5A). Multivariate Cox regression analysis further confirmed the independent prognostic value of ACTN1 expression group, which remained a robust predictor of adverse outcomes (HR = 1.623, *P* < 0.01) (Figure 5B). Based on these results, we constructed a comprehensive nomogram, incorporating relevant indicators, including the ACTN1 expression group, tumor stage, pathological T-staging and pathological N-staging to predict the 1-year, 3-year and 5-year OS of LC patients (Figure 5C). The DCA curve was employed to assess net clinical benefits of the nomogram and individual predictors (Figure 5D). Calibration curves in the training and validation cohorts indicated a favorable agreement between predicted and observed survival probabilities, supporting the predictive efficacy of the nomogram (Figures 5E–H).

A detailed investigation was conducted into the immune characteristics of the ACTN1 high- and low-expression groups. We observed a significant distribution pattern of 28 immune cells and discovered that the majority of tumor-infiltrating immune cells, including activated CD4^+^ T cells, CD56bright natural killer cells, central memory CD8^+^ T cell and so on, were remarkably enriched in high-expression group (Figure 5I). To quantify the immune and stromal components within TME, we calculated the stromal score and immune score for the two ACTN1 expression groups by utilizing “ESTIMATE” algorithm. Box plots displayed the final results, indicating that although patients with higher ACTN1 expression may possess higher stromal score, there was no substantial difference in immune scores between the two subgroups (Figure 5J). Utilizing multiple algorithms for immune infiltration, we validated the aforementioned results, showing that the immune landscape did not exhibit remarkable significance in either group. Notably, cancer-associated fibroblasts were positively correlated with the expression level of ACTN1 (Figure 5K).

Given the pivotal role of immune checkpoint molecules in regulating the TME and shaping the responses to immunotherapy, we compared the expression level of diverse immune checkpoint genes between high- and low-expression groups to inform rational immunotherapy strategies. Notably, the bar plot demonstrated that immune checkpoint-associated proteins, such as CD44, CD80, CD86, CD200, NRP1 and TNFSF4, were actively expressed in the high ACTN1 expression group (Figure 5L). These results suggest that ACTN1-high tumor may potentially benefit from immune checkpoint inhibitors (ICIs).

### 3.6 Exploration and validation of an ACTN1-related signature based on 101 machine learning approaches

**FIGURE 6:**
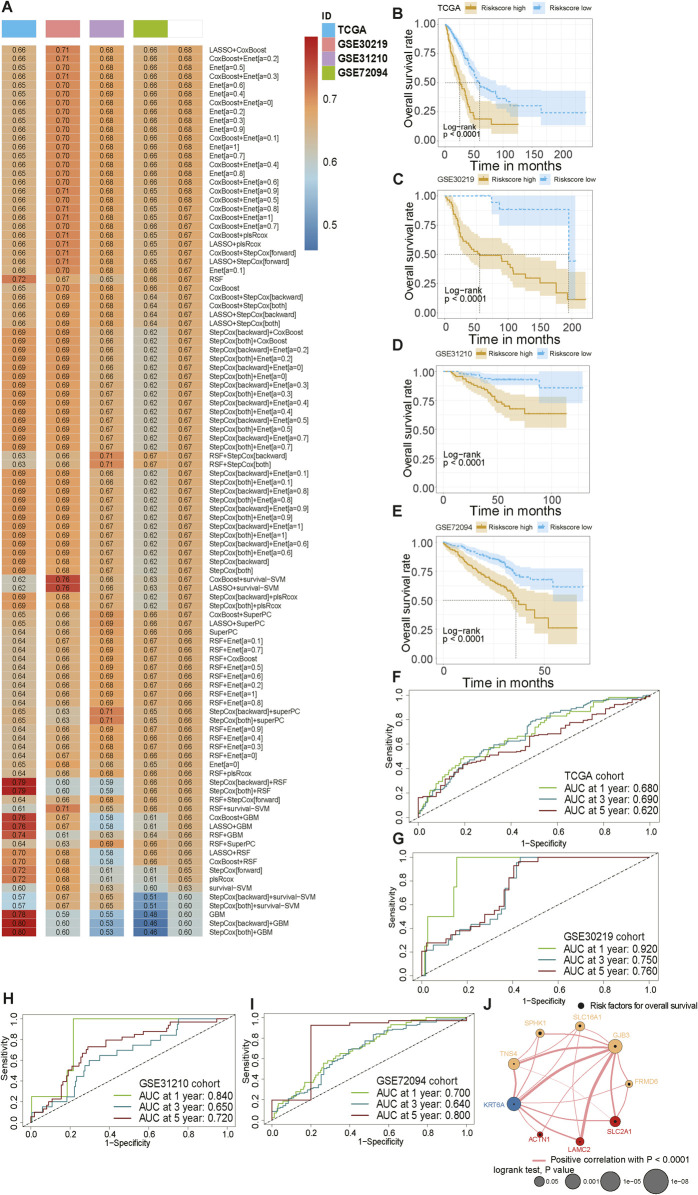
Construction and validation of an ACTN1-related prognostic model. **(A)** Establishment of a prognostic model based on 10 machine learning approaches and their 101 combinations, coupled with ACTN1-related genes; the combination of LASSO and CoxBoost algorithms is identified as the optimal combination. **(B–E)** KM curves manifest the survival differences between populations categorized into high- and low-risk groups. **(F–I)** Receiver Operating Characteristic (ROC) curves are performed to evaluate the discriminatory performance of the model. **(J)** A network illustrates the correlation between ACTN1 and model genes.

Having reinforced the prognostic relevance of ACTN1-related genes, we filtered and obtained 8 key prognostic indicators through survival analysis. Subsequently, TCGA-LUAD dataset was utilized as the training cohort, whereas three external cohorts, including GSE30219, GSE31210 and GSE72094, served as validation cohorts to assess the robustness of the final model. Leveraging training and validation datasets and 10 machine learning approaches (RSF, Enet, Lasso, Ridge, stepwise Cox, CoxBoost, plsRcox, SuperPC, GBM, and survival-SVM), we fitted 101 candidate prognostic models within a LOOCV framework and calculated the average C-index for each model to manifest their performances (Figure 6A).

Judging from the results, the combination of LASSO and CoxBoost algorithms achieved the highest C-index and was therefore selected to construct the ACTN1-associated prognostic signature. Risk score was calculated as follows: Risk score = (FRMD6 * 0.0016) + (GJB3 * 0.1816) + (KRT6A * 0.2812) + (LAMC2 * 0.0012) + (SLC2A1 * 0.1101) + (SLC16A1 * 0.0656) + (SPHK1 * 0.0158) + (TNS4 * 0.0687). The gene coefficients were derived from the results of LASSO regression analysis.

In all cohorts, patients were stratified into high- and low-risk groups according to the optimal cutoff of the calculated risk score, and the signature significantly discriminated survival outcomes (*P* < 0.001) (Figures 6B–E). Time-dependent ROC curves were generated to assess the predictive performance of the prognostic model, and the 1-year, 3-year and 5-year AUCs for different datasets were calculated as follows: TCGA cohort (0.680, 0.690, 0.620), GSE30219 cohort (0.920, 0.750, 0.760), GSE31210 cohort (0.840, 0.650, 0.720), GSE72094 cohort (0.700, 0.640, 0.800) (Figures 6F–I), supporting the robust predictive capacity of the prognostic model. Finally, the correlation network demonstrated the interaction relationship between module genes and ACTN1, illustrating their malignant roles in patients’ prognosis (Figure 6J).

### 3.7 Investigating the expression of ACTN1 in clinical samples

The tumor-promoting function of ACTN1 was suggested by our analyses, and the adverse impact of ACTN1-related genes on the survival outcomes of patients with LCBM was evident. Thus, we initially examined ACTN1 protein expression in clinical specimens retrieved from the HPA database. IHC staining results for ACTN1 were markedly stronger in LC tissues than those in normal tissues (Supplementary Figure S3A), supporting elevated expression of ACTN1 in malignant tissues.

Moreover, we obtained ST data from primary and osimertinib-resistant LC tissues to reveal their tissue architectures. Two pathological images of osimertinib-resistant LC tissues are depicted in Supplementary Figures S3B, C, whereas four corresponding primary LC tissues are shown in Supplementary Figures S3D–G. By eliminating intergroup batch effects and analyzing ACTN1 expression across different specimens after normalization, we discovered higher ACTN1 enrichment in osimertinib-resistant tissues (Supplementary Figures S3H, I) than that in primary specimens (Supplementary Figures S3J–M). Conclusively, these results suggest that ACTN1 may contribute to tumor progression and EGFR-TKI resistance, which warrants further experimental validation.

### 3.8 ACTN1 may drive metastasis and EGFR-TKI resistance in LC cells

To explore the mechanistic association between ACTN1 and the observed poor survival outcomes, particularly in the context of targeted therapies, we first performed a visual KO assay and identified the top 20 most perturbed genes, as shown in a bar plot (Supplementary Figure S4A). By conducting KEGG analysis, we revealed marked alterations in biological functions, including endocytosis, oxidative phosphorylation and regulation of actin cytoskeleton (Supplementary Figure S4B). Additionally, GO analysis of biological process, cellular component, and molecular function indicated that the most altered genes were predominantly involved in epithelial proliferation and migration, endosome and actin binding (Supplementary Figure S4C). Collectively, ACTN1 may play a pivotal role in the proliferation and metastasis by regulating the cytoskeleton architecture. The enhanced metabolic activity may contribute to an increased capacity for EGFR-TKI resistance.

**FIGURE 7:**
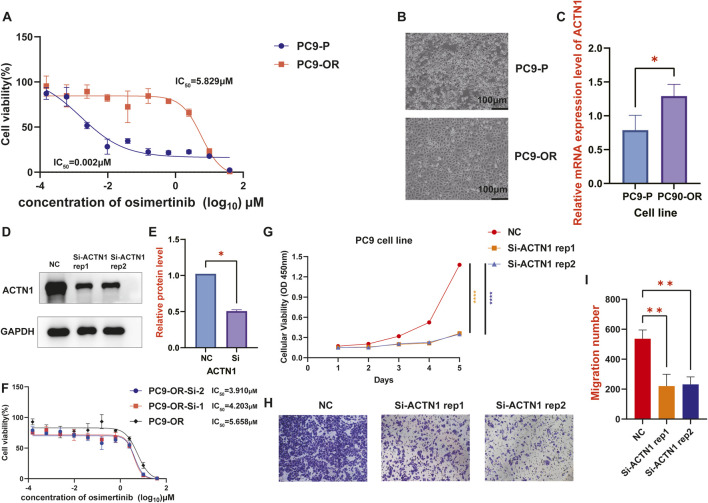
Experimental validation of the function of ACTN1 in inducing EGFR-TKI resistance and malignant phenotypes in the PC9 cell line. **(A)** Osimertinib-resistant and parental PC9 lung adenocarcinoma cell lines (designated PC9-P and PC9-OR) are treated with osimertinib, followed by CCK-8 assays. **(B)** Representative images of PC9-P and PC9-OR cells under a 10× microscope. **(C)** A bar plot reveals the result of qPCR, illustrating relative mRNA expression levels of ACTN1 in PC9-P and PC9-OR cell lines. **(D,E)** Western blotting shows ACTN1-silencing efficiency in PC9-OR cells. Quantification is performed using ImageJ software. **(F)** PC9-OR and ACTN1-silenced PC9-OR cells are treated with osimertinib, and their sensitivities are assessed by CCK-8 assays. **(G)** CCK8-assays are conducted in primary and ACTN1-silenced PC9-OR cells. Cellular viabilities of all cell groups within 5 days are quantified by optical density (OD) values at 450 nm and depicted in a line chart. **(H,I)** Transwell migration assays are conducted in primary and ACTN1-silenced PC9-OR cells. The number of migration cells are counted using ImageJ software. (*p*-value ≥0.05, ns; *p*-value <0.05, *; *p*-value <0.01, **; *p*-value <0.001, ***; *p*-value <0.0001, ****).

To further confirm the function of ACTN1, an osimertinib-resistant cell line of the PC9 lung adenocarcinoma cells (designated PC9-OR) was established, with the parental PC9 cells (designated PC9-P) serving as the control. In response to osimertinib, the IC_50_ value of PC9-OR cell line was significantly higher than PC9-P cell line (IC_50_ for PC9-OR = 5.829 μM versus PC9-P = 0.002 μM) (Figure 7A). Representative images of the cell lines under a 10× microscope are shown in Figure 7B. ACTN1 mRNA levels were quantified by qPCR, confirming a significant increase in ACTN1 expression in the PC9-OR cells compared to the PC9-P cells (*p*-value <0.05) (Figure 7C). We further silenced ACTN1 expression in PC9-OR cells and validated the silencing efficiency by Western blotting (Figures 7D,E). The original Western blot images are provided in Supplementary Figure S5. Drug sensitivity assays confirmed increased sensitivity to osimertinib in ACTN1-silenced PC9-OR cells (Figure 7F). These phenomena strongly suggest that the upregulation of ACTN1 may contribute to the acquisition of EGFR-TKI resistance. In addition, the downregulated expression of ACTN1 expression weakened the migratory and proliferative capacity of osimertinib-resistant LC cells, supported by cell viability (Figure 7G) and Transwell assays (Figures 7H,I).

## 4 Discussion

Referring to previous studies, a substantial body of reliable evidence indicates that LCBM is a common complication in patients with LC, and is associated with poor prognosis, which prompted us to investigate this condition further (Duan et al., 2024; Zuccato et al., 2025). Numerous hypotheses have been proposed to elucidate the underlying mechanisms, with most concentrating on the transformation of TME and immune evasion (Wang X. et al., 2024; Xiao Y. et al., 2024; You et al., 2024). Epithelial cells, as a pivotal and predominant cell population, exhibit significant heterogeneity and plasticity, and play a crucial role in the initiation and progression of malignant phenotypes (Han et al., 2024; Marjanovic et al., 2020). On one hand, various biological processes can reshape the TME, thereby facilitating metastasis and resistance to targeted therapies, especially EGFR-TKIs (Ch et al., 2022; Hung et al., 2024; Su et al., 2020). On the other hand, LC cells that acquire resistance to EGFR-TKIs are frequently enriched in brain lesions, potentially establishing a poised state that contributes to refractory outcomes (Fu et al., 2024; Adua et al., 2022). Thus, we performed a combined single-cell analysis of LCBM lesions, with a specific focus on the heterogeneity of epithelial cells.

The acquisition of brain metastatic tissues from patients with acquired resistance to EGFR-TKIs is constrained by ethical and practical limitations. Therefore, we initially utilized “Beyondcell”, an algorithm capable of predicting cellular responses to diverse agents, to explore the drug-response heterogeneity among epithelial cells (Zhang et al., 2024). The Semi-Supervised Optimization of Rare-Cell Silhouettes (SCISSORS) approach was then used to identify and characterize phenotype-associated cell populations within scRNA-seq data, enabling us to detect cells of extremely low abundance (Leary et al., 2023; Lv et al., 2022; Ye et al., 2025). After observing marked differences in predicted drug response among distinct clusters of epithelial cells via SCISSORS analysis, we subsequently uncovered hub genes within resistant clusters and ultimately pinpointed ACTN1 as a critical factor contributing to therapeutic resistance.

As a member of the α-actinin family, ACTN1 is associated with cytoskeleton remodeling and has been regarded as a tumor-promoting factor in various malignancies. Firstly, in ovarian cancer, ACTN1 induces actin filament aggregation and the polarization of M2-like macrophages, which collectively foster an immunosuppressive and highly proliferative TME that is conducive to invasion and progression (Gu et al., 2023; Sun et al., 2025). Additionally, ACTN1 modulates the TME and interacts with other factors to promote malignant behaviors, including proliferation, transformation, metastasis and colonization (Chen et al., 2024; Kovac et al., 2018). From another perspective, ACTN1-mediated cytoskeletal remodeling also contributes to therapy resistance in head and neck squamous cell carcinoma, triple-negative breast cancer and hepatocellular carcinoma (Cui et al., 2023; Inayatullah et al., 2024; Xiao H. et al., 2024). Nonetheless, there is a paucity of studies exploring the relationship between ACTN1 and LC, with only one clinical experiment suggesting a correlation between ACTN1 and with tumor stages, aligning with lymph node metastasis (Hirooka et al., 2011). To bridge this gap, we further inferred developmental trajectory within the single-cell atlas and found that ACTN1^+^ epithelial cells were positioned at a differentiated state. These cells may potentially possess the capacity to recruit substantial numbers of granulocytes and remodel the existing TME, as inferred from dynamic functional analyses. Concurrently, LR and signaling pathway analyses demonstrated elevated levels of COLLAGEN, MIF and MK signaling pathways. Based on previous studies, we hypothesize that ACTN1^+^ epithelial cells may, on one hand, induce the production of collagen matrix to facilitate angiogenesis, cell migration and therapy resistance (Wang L. et al., 2024; Cescon et al., 2023; Vymola et al., 2024). On the other hand, they may recruit neutrophils to promote T cell exhaustion and suppress macrophage function, collectively establishing an immunosuppressive TME favorable for tumor cell proliferation (Sun et al., 2024; Yuan et al., 2016; Liu et al., 2024).

From a transcriptome perspective, clear classification based on the expression levels of ACTN1 was observed across various LC datasets. Notably, IL-17 and PI3K-Akt signaling pathways were upregulated in the ACTN1-high subgroup, accompanied by enhanced ECM organization and chemokine signaling. Relevant literature illustrates these enriched pathways play significant roles in epithelial-mesenchymal transition (EMT) (Yuan et al., 2022; Xie et al., 2022; Inthanon et al., 2023). First, EMT is characterized by the loss of epithelial features and the acquisition of mesenchymal traits, enabling enhanced cell invasion and motility (Fontana et al., 2024; Chen et al., 2019). In our analyses, the upregulation of PI3K-Akt signaling, together with increased ECM organization, is consistent with an EMT-permissive microenvironment. Previous studies also demonstrate specific capacity of PI3K-Akt axis to inhibit autophagic flux and lead to metastasis of LC cells by enhancing EMT signaling pathway (Xu et al., 2024; Lu et al., 2017; L et al., 2021; Li et al., 2021). Furthermore, inflammatory signaling driven by IL-17 has been reported to promote EMT through cytokine induction and phosphorylation of Akt, JNK, ERK1/2, and STAT3 (Xie et al., 2022; Inthanon et al., 2023; Huang et al., 2016). The coordinated activation of these two signaling pathways in ACTN1-high tumors may facilitate cell detachment, motility, and metastatic colonization, thereby reinforcing EMT-associated progression.

Through stratifying all patients into distinct groups based on the expression level of ACTN1 and constructing predictive models, we validated the prognostic value of ACTN1 for patients’ survival outcomes. Additionally, we performed a detailed characterization of the TME across different groups, revealing that a generally activated stromal microenvironment and a relatively preserved immune profile were consistent with the aforementioned findings. A 9-gene ACTN1-related signature was further developed and demonstrated high predictive efficiency across multiple datasets, as supported by the C-index and AUC values. Although most of these genes have been previously associated with malignant phenotypes of LC cells, GJB3 was specifically identified as a hub gene that modulates sensitivity to EGFR-TKIs in LUAD and aids in identifying potential beneficiaries of immunotherapy (Lin et al., 2024; Wang and Zhang, 2025). Its clinical utility and potential interaction with ACTN1 warrant further investigation.

In our analysis, the “Beyondcell” algorithm was used to screen drug-response tendencies across cell subpopulations using available perturbation signatures. Prediction results suggested that ACTN1-high (Scissor^+^) epithelial programs are associated with a transcriptional state of EGFR-TKI resistance. Therefore, we selected osimertinib for experimental validation instead of gefitinib or afatinib, as it is a representative third-generation EGFR-TKI widely used in contemporary clinical practice, including as first-line therapy for EGFR-mutant LC patients (Ramalingam et al., 2020; Soria et al., 2018) and in the setting of acquired resistance to earlier-generation EGFR inhibitors (Cross et al., 2014; Moores et al., 2016). Establishing an osimertinib-resistant model not only improves the clinical relevance of our research, but also allows us to test whether ACTN1 contributes to EGFR-TKI resistance under a more current therapeutic condition. Utilizing expression assays, we validated the upregulation of ACTN1 in PC9-OR cells. Furthermore, silencing ACTN1 restored drug sensitivity and reduced migratory capacity, supporting that ACTN1 is involved in EGFR-TKI resistance and metastatic phenotypes consistent with purely computational prediction.

Ultimately, it is essential to acknowledge several limitations of our study. Although we confirmed elevated ACTN1 expression in ST data from clinical samples and in a constructed osimertinib-resistant cell line, the efficacy of specifically targeting ACTN1 to reverse the EGFR-TKI resistance and treat LCBM requires validation in large-scale clinical trials. Additionally, the malignant role of ACTN1 and its downstream regulatory mechanisms, including its association with EMT, require further elucidation using advanced experimental methods. Given the potential biases and challenges associated with sample collection, we aim to refine our single-cell studies by incorporating a broader array of clinical specimens. This effort is expected to pave new pathways for the treatment of patients with LCBM and those experiencing EGFR-TKI resistance.

## 5 Conclusion

In summary, our comprehensive research profiled the single-cell landscape of LCBM specimens and identified ACTN1 as a key factor associated with the development of EGFR-TKI resistance and malignant phenotypes. We further constructed an ACTN1-related gene signature as a prognostic biomarker and a source of candidate therapeutic targets for patients with LCBM. Concisely, our study pioneers a fresh perspective on investigating LCBM and its drug-resistance traits, addressing a previously challenging research area, and identifying potential effective therapeutic targets for long-term management.

## Data Availability

The original contributions presented in the study are included in the article/Supplementary Material, further inquiries can be directed to the corresponding authors.

## Supplementary material

SUPPLEMENTARY FIGURE S1 Identification of 487 genes associated with EGFR-TKI resistance. The GSE231938 and GSE262582 datasets were utilized.

SUPPLEMENTARY FIGURE S2 A forest plot depicting the survival significance of genes in models correlated with EGFR-TKI resistance, by employing univariate Cox regression analysis.

SUPPLEMENTARY FIGURE S3 The ACTN1 expression landscape in lung cancer tissues. **(A)** Immunohistochemistry (IHC) staining results indicating the expression levels of ACTN1 in lung cancer and normal tissues. **(B,C)** Spatial transcriptomic (ST) plots of two osimertinib-resistant lung cancer tissues. **(D–G)** ST plots of four primary lung cancer tissues. **(H,I)** ST plots showing the expression of ACTN1 within two osimertinib-resistant lung cancer tissues. **(J–M)** ST plots showing the expression of ACTN1 within four primary lung cancer tissues.

SUPPLEMENTARY FIGURE S4 Visual knockout assays performed on ACTN1 within epithelial subclusters. **(A)** The top 20 differentially perturbed genes after ACTN1 knockout are visualized in a bar plot. **(B)** KEGG analysis performed on the most perturbed genes. **(C)** GO analysis performed on the most perturbed genes.

SUPPLEMENTARY FIGURE S5 Original images of Western blotting, reflecting the silencing effect of ACTN1 protein, with GAPDH as the internal control.
