# Supplementary figures and images for: Integrative single-cell and bulk RNA sequencing unravels the role of ACTN1 in promoting lung cancer with brain metastasis and epidermal growth factor receptor-tyrosine kinase inhibitor resistance
Source: Front Cell Dev Biol. 2026 Apr 14;14:1738641. doi: 10.3389/fcell.2026.1738641 (PMC13122775; doi:10.3389/fcell.2026.1738641)

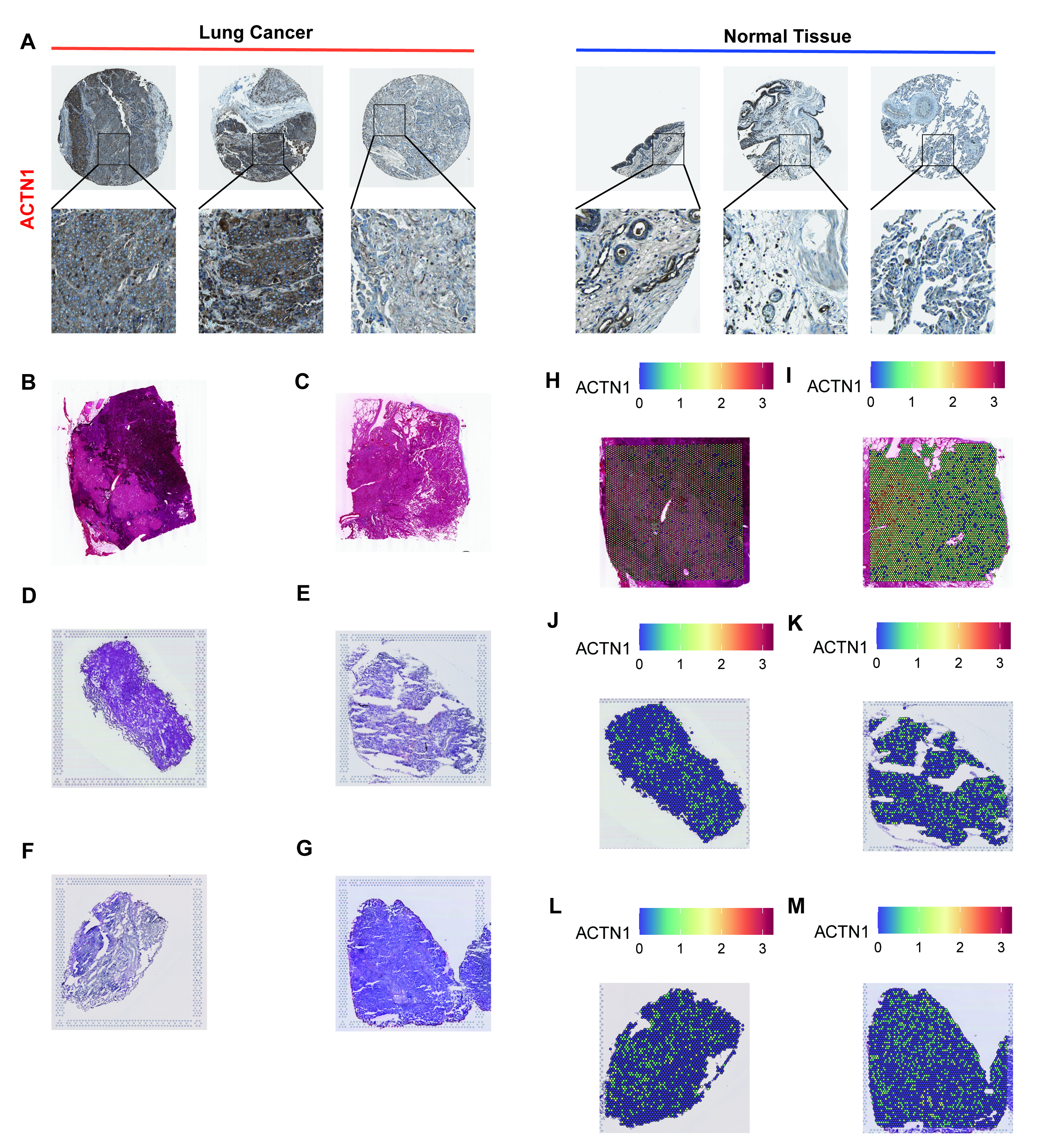

Supplement: Supplementary file 1 [file Image3.tif]

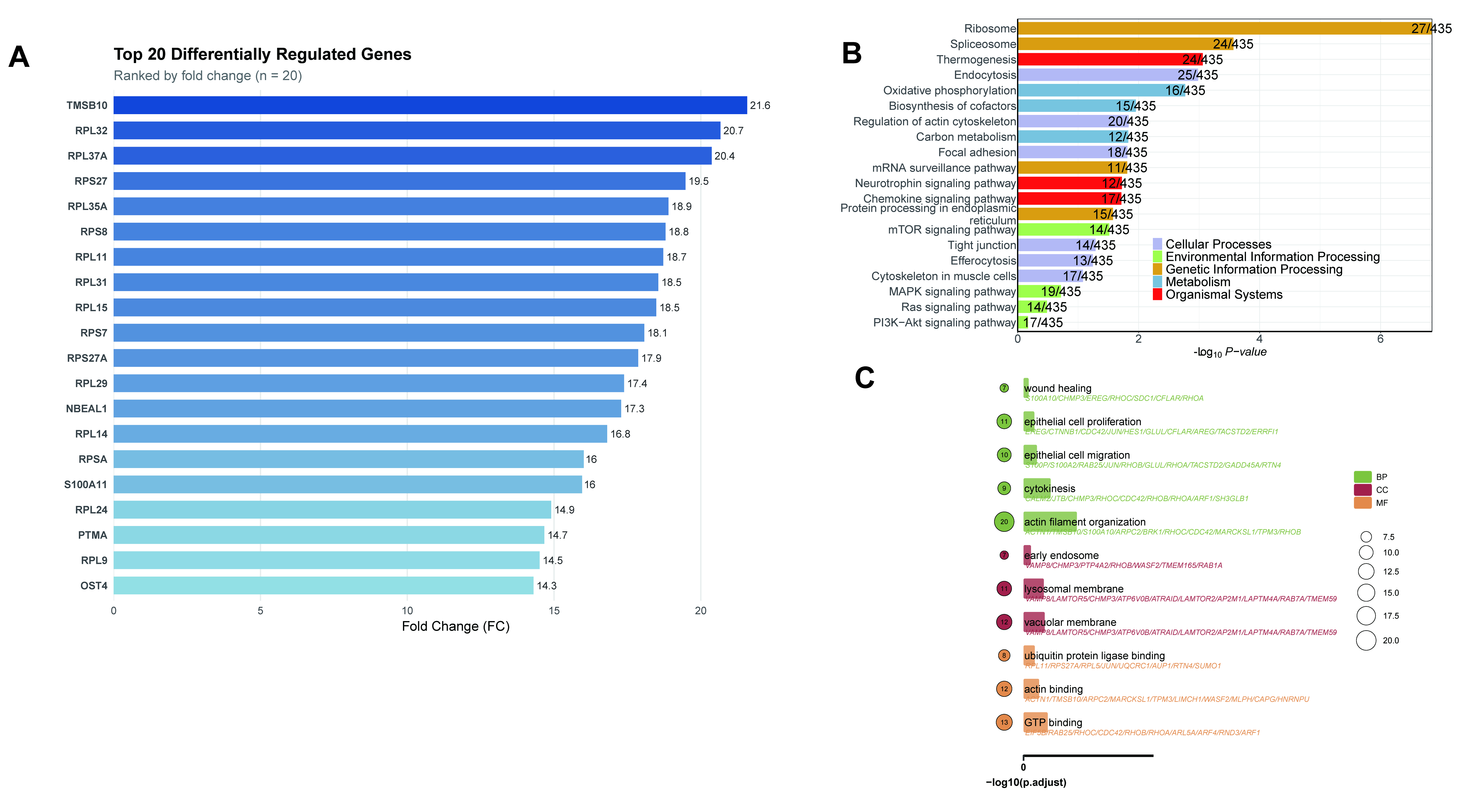

Supplement: Supplementary file 2 [file Image4.tif]

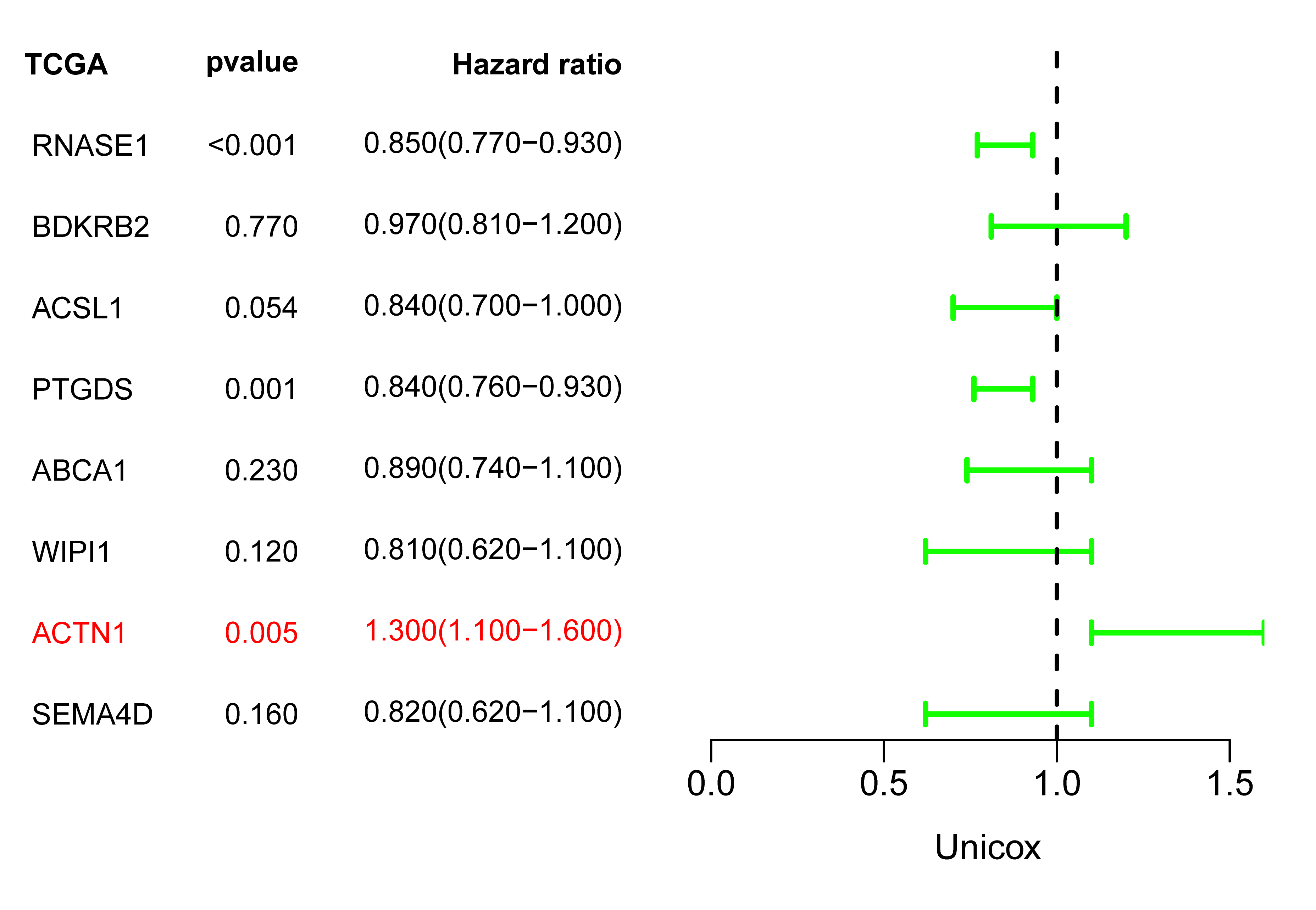

Supplement: Supplementary file 3 [file Image2.tif]

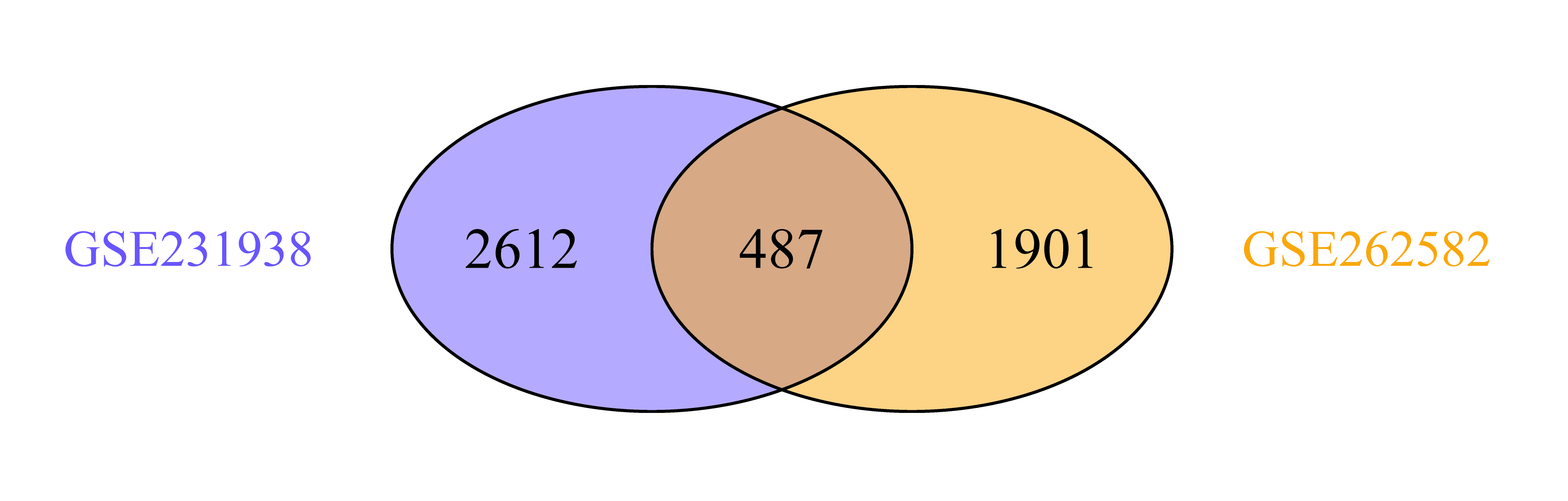

Supplement: Supplementary file 4 [file Image1.tif]

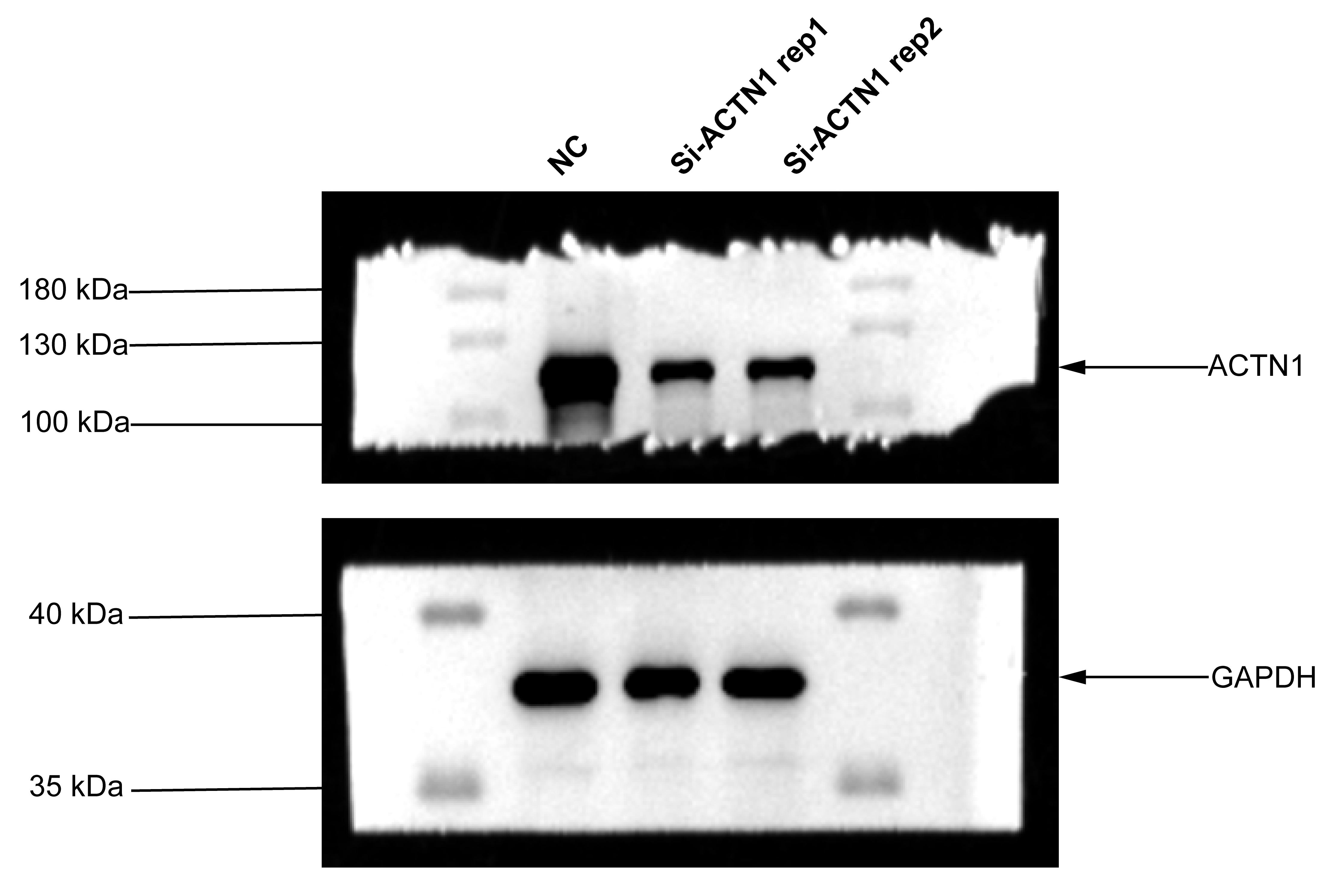

Supplement: Supplementary file 5 [file Image5.tif]
